# Titanium Wear of Dental Implants from Placement, under Loading and Maintenance Protocols

**DOI:** 10.3390/ijms22031067

**Published:** 2021-01-21

**Authors:** Georgios E. Romanos, Gerard A. Fischer, Rafael Delgado-Ruiz

**Affiliations:** 1 Department of Periodontology, Laboratory for Periodontal-, Implant-, Phototherapy (LA-PIP), School of Dental Medicine, Stony Brook University, 106 Rockland Hall, Stony Brook, NY 11794-8700, USA; gerard.fischer@stonybrookmedicine.edu; 2 Department of Oral Surgery and Implant Dentistry, School of Dentistry, Johann Wolfgang Goethe University, 60590 Frankfurt, Germany; 3 Department of Prosthodontics and Digital Technology, School of Dental Medicine, Stony Brook University, Stony Brook, NY 11794-8700, USA; rafael.delgado-ruiz@stonybrook.edu

**Keywords:** nanoparticles, peri-implantitis, titanium

## Abstract

The objective of this review was to analyze the process of wear of implants leading to the shedding of titanium particles into the peri-implant hard and soft tissues. Titanium is considered highly biocompatible with low corrosion and toxicity, but recent studies indicate that this understanding may be misleading as the properties of the material change drastically when titanium nanoparticles (NPs) are shed from implant surfaces. These NPs are immunogenic and are associated with a macrophage-mediated inflammatory response by the host. The literature discussed in this review indicates that titanium NPs may be shed from implant surfaces at the time of implant placement, under loading conditions, and during implant maintenance procedures. We also discuss the significance of the micro-gap at the implant-abutment interface and the effect of size of the titanium particles on their toxicology. These findings are significant as the titanium particles can have adverse effects on local soft and hard tissues surrounding implants, implant health and prognosis, and even the health of systemic tissues and organs.

## 1. Introduction

Dental implants have become a popular option for the replacement of one or more missing teeth in the oral cavity due to their long-term success rate [1]. Almost 11% of dental implants fail and are subsequently removed within the first 15 years of placement and osseointegration [2]. Regardless of the etiology, the point at which an implant becomes mobile is the point where it has become a failed implant. Bacterial challenge to the peri-implant tissues, in the form of plaque biofilms, is a well-known cause of local inflammation that may eventually lead to peri-implant mucositis, and in some patients, peri-implantitis [3]. Peri-implant biofilms differ in composition than those associated with teeth. Peri-implant biofilms contain some unique species as well as bacterial forms associated with periodontal pockets [4]. The peri-implant tissue is described as a zone approximately 1mm thick surrounding the implant body where interactions with peri-implant bone may occur [5,6]. Peri-implantitis is an inflammatory condition of the supporting bone around a dental implant. Peri-implant mucositis, which generally precedes peri-implantitis, is reversible inflammation of the soft tissue surrounding an implant [7,8]. The clinical signs of peri-implant conditions include bleeding on probing, suppuration, and increased probing depths. The diagnosis is confirmed by radiographic peri-implant bone loss [9].

Recently, studies on biomaterials have become an important part of dental implant research as investigators attempt to better understand the etiology of implant failures. Manufacturers have developed numerous different implant designs differing in proportions of metals and other materials as well as surface roughening treatments all in an effort to improve osseointegration and biocompatibility while decreasing the rate of implant failure [10]. Some of these implants include commercially pure (c.p.) titanium, Titanium Aluminum Vanadium (Ti-6Al-4V) alloys, and recently, titanium-zirconia alloys [11,12]. They are supposedly highly biocompatible and resistant to corrosion due to their ability to form a passive oxide film following exposure to oxygen [12].

Manufacturers have also modified the surface roughness of implants to improve the osseointegration by acid etching, sandblasting, and oxidation techniques [13]. Extensive research concluded that pure titanium and titanium alloys are both highly biocompatible with low toxicity and favorable properties for osseointegration [14]. Many of these studies, however, do not focus on the properties of dental implant materials when they are broken down to smaller particles. There has been growing concern over this issue as newer studies have shown that there are many phases of an implant’s life cycle during which there is wear of the implant that leads to the shedding of titanium particles which are then introduced to the local tissues [2]. Once the titanium particles are shed from the implant surface, they can induce local inflammation. They may also be transported away from the oral cavity, after which the particles can be found, causing inflammation in distant tissues, with potential systemic involvement [14].

The aim of this review was to summarize the current knowledge related to titanium wear produced during implant placement, loading, and maintenance with special focus in the biophysiological interactions at the implant abutment interface. Additionally, the mechanisms by which titanium particles are taken up by cells and induce inflammation will be discussed. Finally, the significance of the size of the particles, and the possible systemic effects the titanium particles can cause following ingestion from the oral cavity will also be evaluated.

## 2. Titanium Wear at the Time of Implant Insertion

The study of titanium particles liberated from dental implant surfaces has become an important area of research in dental implantology. A 1993 study conducted by Schliephake et al. revealed the presence of titanium in the adjacent soft tissue surrounding titanium screws used to treat jaw fracture. The titanium was viewed 5–8 months post-operatively in transmission electron microscope images. The study suggested that the titanium particles localized extracellularly may be taken up by cells for lysosomal degradation, and the remnants may be left in place after the macrophages eventually clear out [15]. Subsequent research has found that titanium particles may be shed into the surrounding peri-implant tissues as early as the time of initial implant placement [2,16]. In 2004, Franchi et al. found titanium granules separated from the implant surface in the peri-implant tissues (mucosa and bone) as early as 12 weeks after placement [16]. Their finding implies that masticatory forces, corrosion, and fretting are not necessary for titanium wear to occur and confirms that titanium wear occurs by the friction created between the implant and the bone during the implant insertion [16].

**Figure 1:**
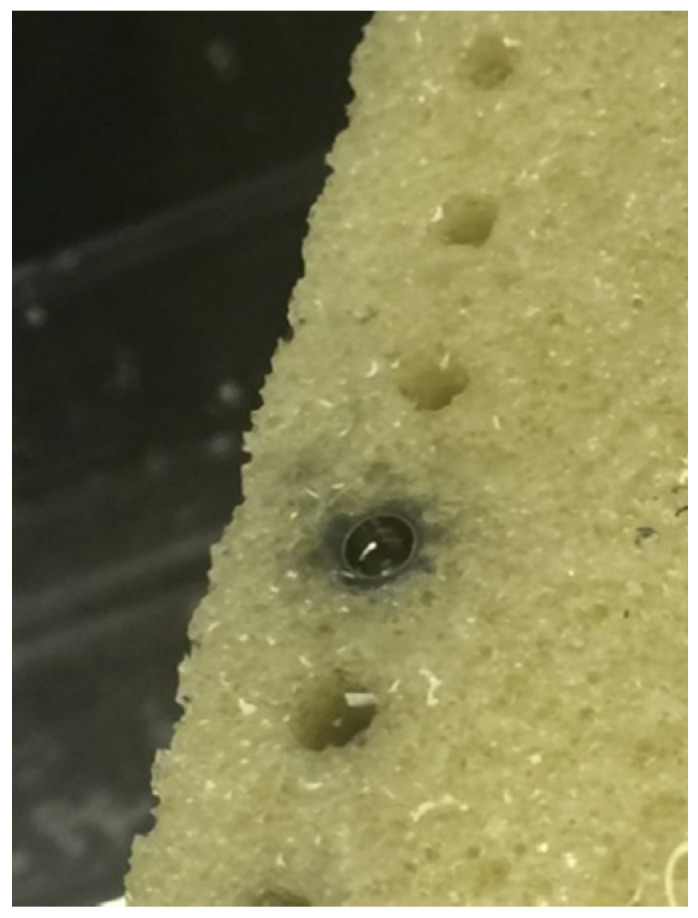
Metal particle apposition in bone block in vitro immediately after surgery.

**Figure 2:**
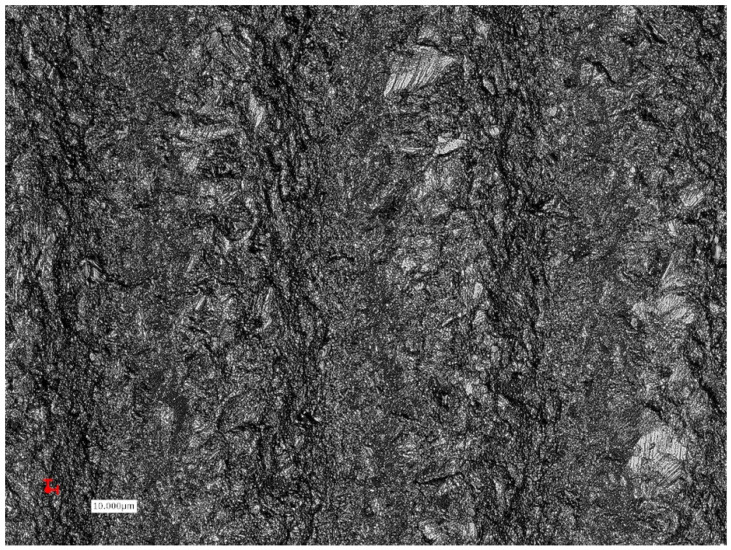
Sandblasted and acid-etched surface demonstrating changes of the implant roughness after implant insertion, and titanium particles detachment from the implant surface (lighter areas).

**Figure 3:**
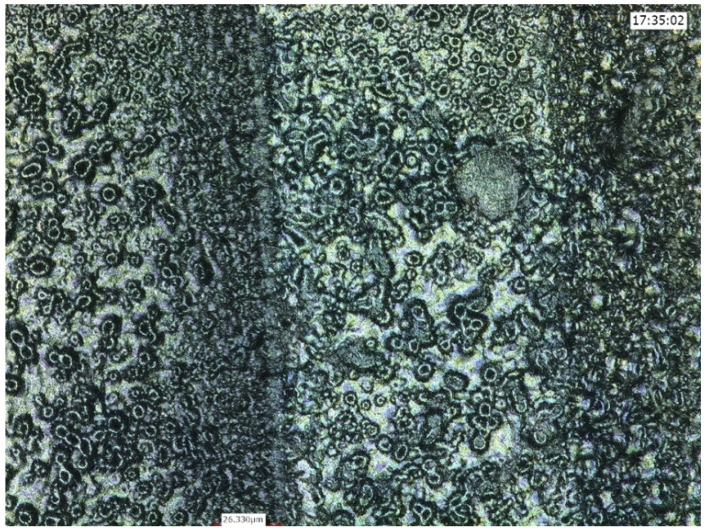
Anodized implant surface demonstrating areas of flattening and disappearance of the typical “crater” appearance, meaning particle detachment (lighter areas).

**Figure 4:**
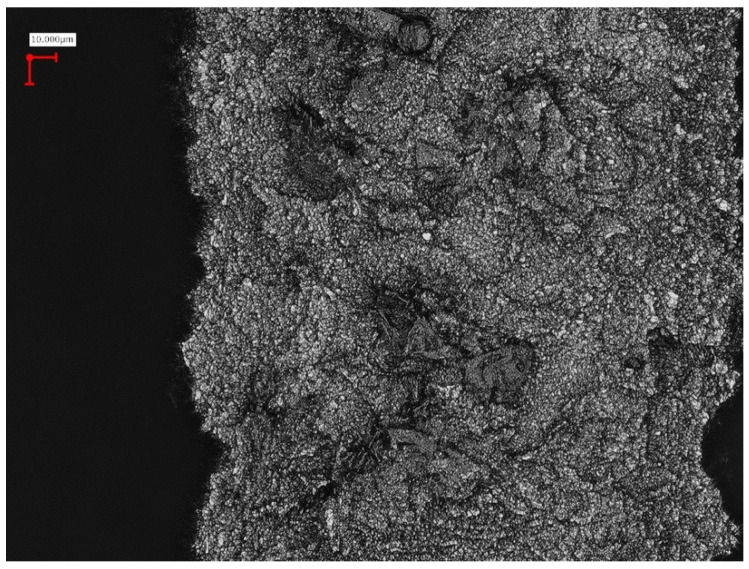
Sandblasted, large grit, acid-etched implant surface (SLA) demonstrating areas of particle detachment, and topographic changes after insertion (darker areas).

**Figure 5:**
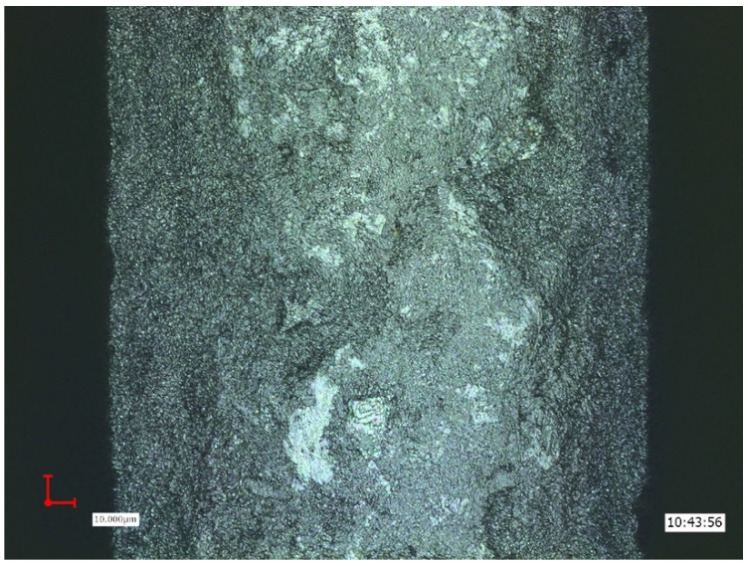
Sandblasted implant surface demonstrating areas of change after insertion.

These results are further supported by a study done by Palazzo et al. [17]. Their histological analysis showed metal particles and ions released from osteosynthesis implants and concluded that metal microparticles could be released during the actual fixation of the implant into the bone (Figure 1, Figure 2, Figure 3, Figure 4 and Figure 5), with the highest concentration of metal found close to the osteosynthesis or implant fixation and as far as 2.5cm away from the implants [17].

Similar findings are seen in recent microscopical EDX analyses of the peri-implant tissues of immediately loaded implants placed in the maxilla and mandible. In an autopsy report conducted 7.5 months after implant placement and loading, analyses showed titanium particles in direct contact to the implant and in distant areas from the implant-bone interface [18].

## 3. Titanium Wear under Conditions of Loading/Forces of Mastication

**Figure 6:**
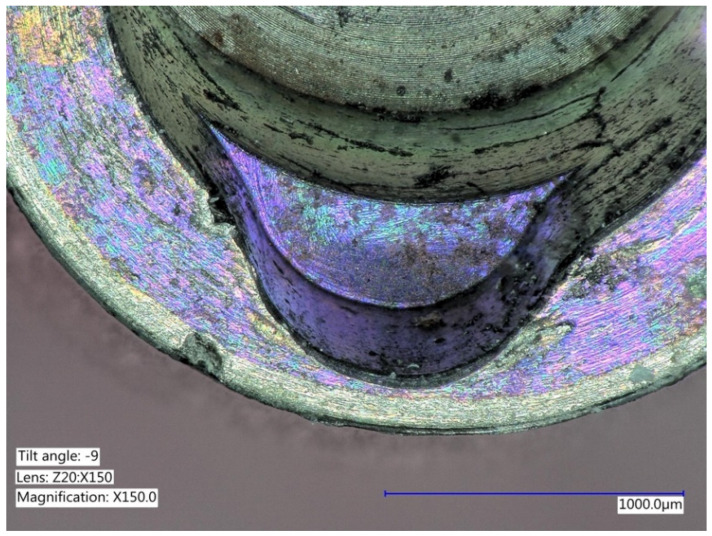
Explanted implant with a Trilobed connection. After disconnection of the implant crown, the evaluation of the implant connection shows severe wear, abrasion of the implant platform, and titanium particles.

**Figure 7:**
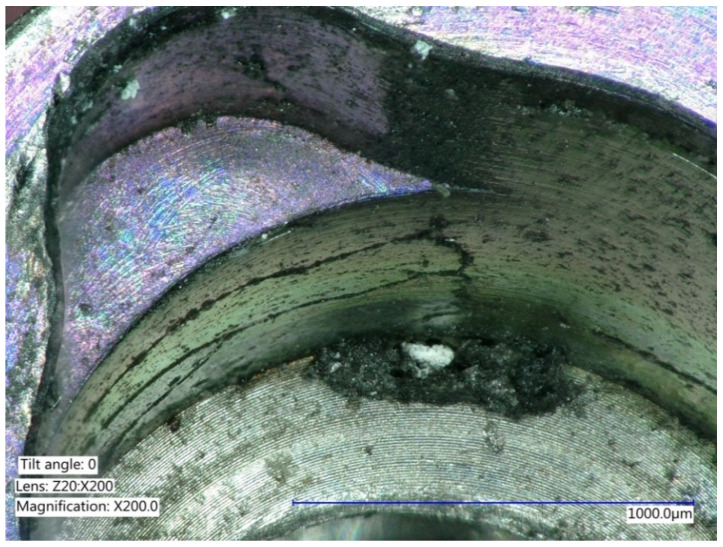
Explanted implant with a Trilobed connection. Increased magnification allows to observe fracture lines, corrosion, and accumulation of titanium particles potentially from implant-abutment connection wear.

**Figure 8:**
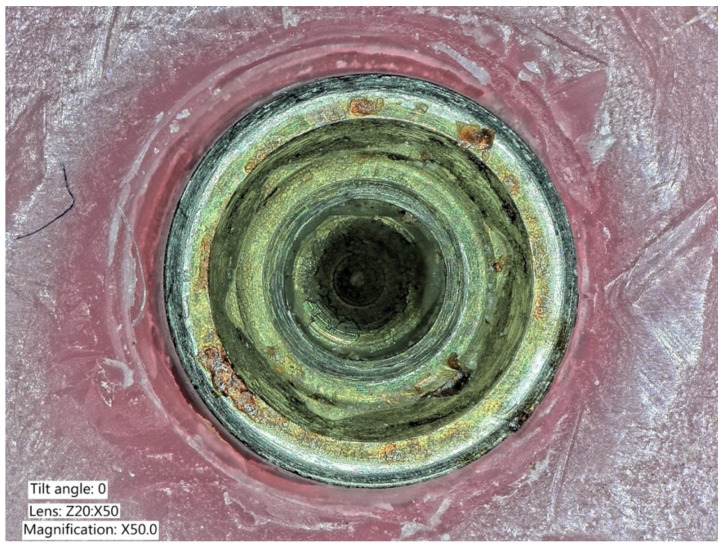
Explanted implant with a hexagonal connection. The implant crown was removed. The implant connection at microscope evaluation showed wear, signs of corrosion, and titanium particles.

**Figure 9:**
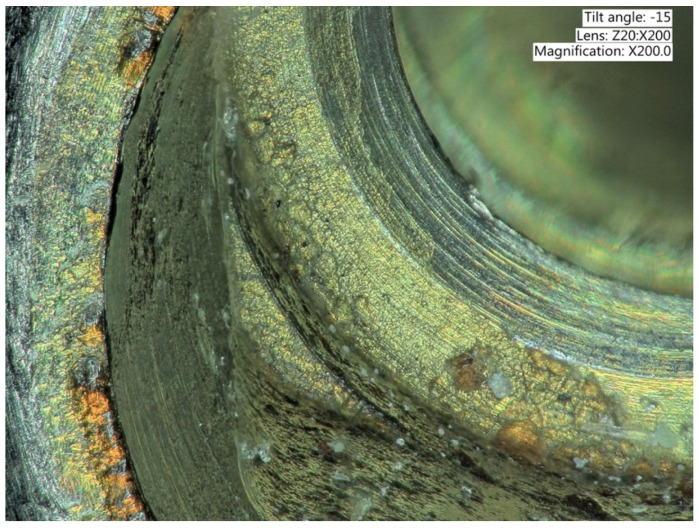
Closer view of an explanted implant with a hexagonal connection. Corrosion, delamination, and abrasions of the connection surface can be appreciated.

**Figure 10:**
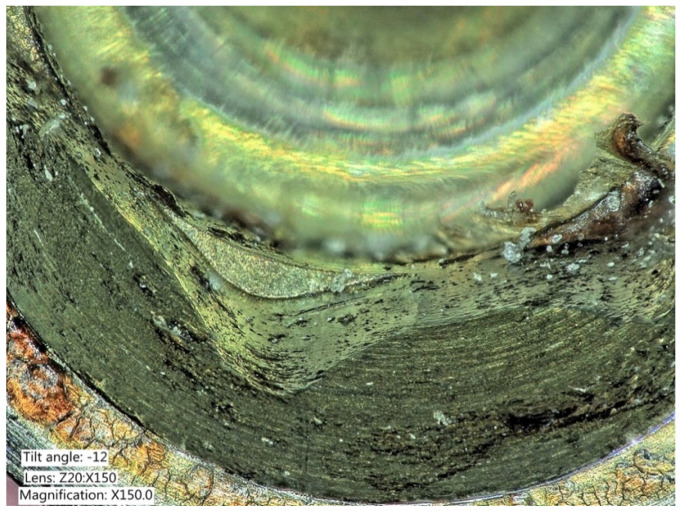
Closer view of an explanted implant with a hexagonal connection. Corrosion, delamination, and abrasions of the connection surface can be appreciated.

One of the most important factors affecting the longevity of a dental implant restoration, is the fit of the implant with the abutment [19]. A less than ideal fit will lead to a larger micro gap between the implant and abutment, where microorganisms, oral fluids, and glycoproteins can accumulate and form stable biofilms leading to corrosion of the material (Figure 6, Figure 7, Figure 8, Figure 9 and Figure 10) [19,20,21,22].

A poor fit with a large micro gap will leave the implant vulnerable to structural damage in the form of fretting and micromovements at the connection when subjected to forces of mastication [19]. In this context, the biofilms act as a lubricant in the connection [19]. As the material at the interface is worn down, the micro gap becomes larger leading to further destabilization of the implant [23]. Morse taper connections have been found to provide a better fit than non-conical connections, thus decreasing the wear of the implant during mastication [24]. Multiple studies show bacteria accumulation in the peri-implant mucosa of implants with external connections, but recent clinical studies comparing morse-tapered with internal polygonal connections demonstrate the accumulation of bacteria deep within the internal (butt-joint) interface compared to the Morse tapered connections, where a bacterial sealing is found [25,26,27,28]. The micromovements of the abutment within the implant body under loading, which is known as “fretting”, leads to titanium nanoparticles in the surrounding implant-tissue interface and further metal corrosion. Studies have shown increased metal debris in the surrounding tissue of peri-implantitis sites compared to the tissue surrounding periodontitis sites. This indicates that metal debris in peri-implant hard and soft tissues may be an etiological factor for peri-implant disease [29]. Recent studies emphasized the importance of the stability of the implant-abutment connection when it comes to the health of peri-implant tissues. It has been found that conical implant-abutment connections minimize the micro gap at the connection than butt-join implant-abutment connections, and conical implant abutment connections allow less bacterial accumulation [30,31]. When the loading forces are increased, the contact wear becomes severe promoting microcracks and particle detachment at the titanium-bone interface especially in the cortical bone and areas with lower content of calcium and phosphate [32]. The significance of increased loading forces is also seen during both osteotomy and manual condensation, where bone density is altered. It has been found via histological analysis that functional loading forces further increase bone density [33,34,35,36].

## 4. Titanium Wear at Implant-Abutment Interface and Impact on the Peri-Implant Tissues

To understand the significance of the interactions at the implant-abutment interface, it is important to understand the phenomena of corrosion and fretting. Corrosion is a process by which a refined metal becomes a more stable oxide, hydroxide, or sulfide. Pure titanium becoming titanium dioxide NP in the peri-implant environment is an example of this conversion. Fretting is the process of wear at the contact points of uneven surfaces. This process occurs under loading forces and may also occur in the presence of corrosion [37]. Fretting refers to wear and sometimes corrosion damage at the asperities of contact surfaces. This damage is induced under load and in the presence of repeated relative surface motion, as induced for example by vibration [37].

**Figure 11:**
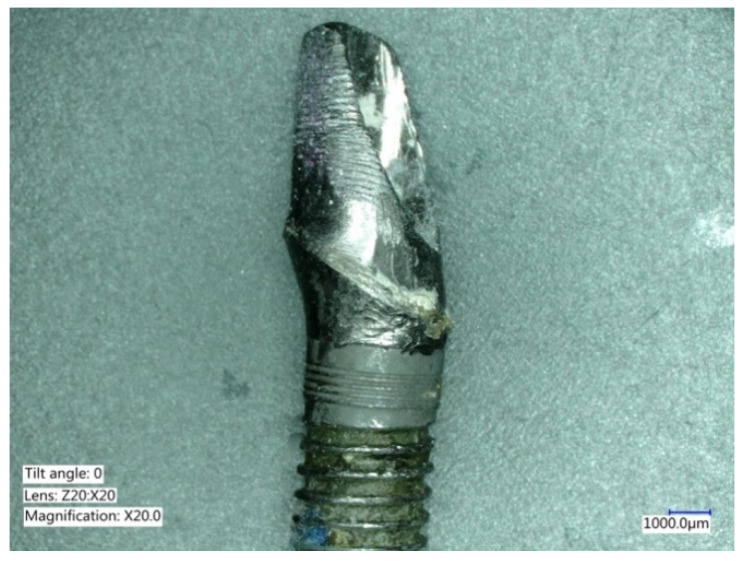
The evaluation of the removed crown abutment, also showed titanium particles and titanium delamination. Surface changes were also evident at the transmucosal portion of the abutment.

**Figure 12:**
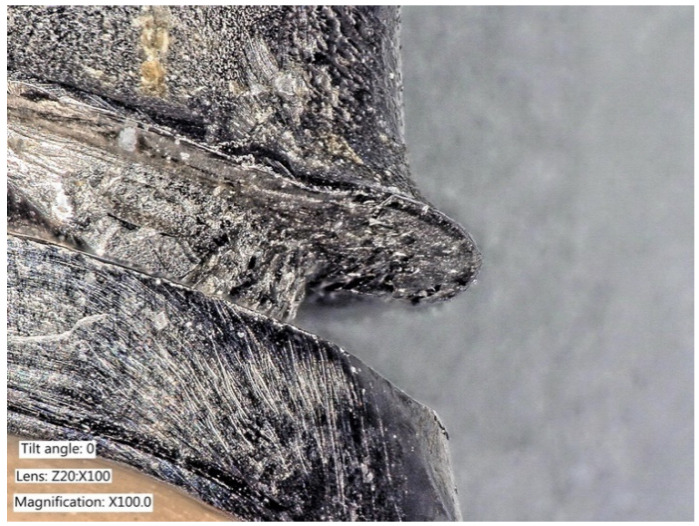
Corrosion of the abutment can occur and appears as pits, or color changes (dark, brown or yellow) distinguishable from the natural gray color of the implant.

**Figure 13:**
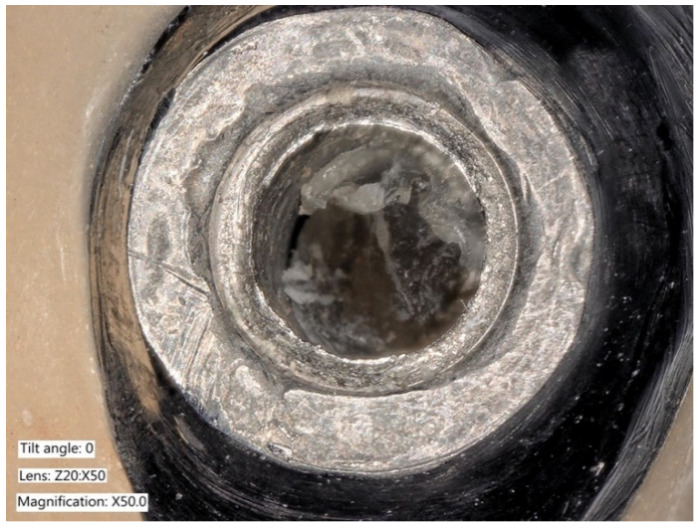
Evaluation of the basal aspect of the implant crown, showing deformation of the hexagon, and particle delamination.

There has recently been an observation of increased titanium dissolution products in biofilms isolated from peri-implantitis cases. These results suggest a possible modification in the structure and diversity of the microbiome due to the titanium dissolution [38]. These findings led to the investigation of the mechanism by which titanium disintegration occurs and factors that may play a role. Additionally, the role of titanium corrosion has become an area of interest [39]. Titanium implants are said to be resistant to corrosion; however, studies have shown that the stable oxide layer that is formed during the process of osseointegration is lost under certain conditions, particularly those seen in the oral cavity. Once this layer is lost, biodegradation and corrosion of the implant may occur [40,41]. Therefore, while titanium implants may be resistant to corrosion in vitro, this property should be explored more in depth in the oral environment [42]. Recent studies have shown that the acidic metabolic by-products of microorganisms and some substances found in fluoride solutions may increase corrosion (Figure 11, Figure 12 and Figure 13), especially at the implant-abutment interface, where the micro gap exists [19,43,44,45,46,47]. In general, significant inflammatory conditions decrease titanium’s resistance to corrosion [12]. This is a significant area of concern in dental implant research as titanium corrosion can contribute to peri-implantitis and eventual implant failure [48].

The corrosion of titanium is enhanced by the presence of H_2_O_2_ and albumin [49,50,51]. Inflammation is associated with production of highly reactive chemical molecules formed due to the electron acceptability of O_2_, so called reactive oxygen species (ROS), such as H_2_O_2_ and superoxide ions, which further increase the corrosion of titanium metal. The inflammatory state is associated with increased neutrophils and macrophages which generate ROS via the respiratory burst pathway [52]. This phenomenon leads to higher porosity of the oxide films and a formation of a rougher, thicker implant surface. In general, the inflammatory state is associated with an acidic environment. This is due to increased oxygen and energy demands of inflammatory cells in the tissue. Glucose is metabolized at a greater rate via glycolysis; thus, more lactic acid is generated [53]. Metal degradation, in conjunction with the acidic environment of inflammation, concentrates the ROS species and up-regulates the corrosion of the implant body, thus promoting the accumulation of titanium ions and particles in the surrounding tissues with more bone resorption and loosening of the implant [54].

Surface analysis of retrieved titanium alloy hip implants from humans were investigated for evidence of localized or general corrosion in modular interfaces when mechanical abrasion of the oxide film (fretting) occurs. The study showed cracking, etching, pitting and delamination of the surface, as well as degradation in the crevice environment during fretting-crevice corrosion and hydrogen embrittlement [55]. Two surfaces under sliding contact induce formation of debris leading to more abrasion and creating a third body particles with impact on the inflammatory reaction in the peri-implant tissues as a process of bio-tribocorrosion [56,57,58,59,60,61].

Titanium accumulation of 13 ppm and above has been shown to induce epithelial cell necrosis and increase the sensitivity of epithelial cells of the peri-implant mucosa in microorganisms. This observation is due to ROS production and increased cytokine levels, leading to a reduction of cell viability and proliferation. Additionally, the induction of apoptosis and genotoxicity have been documented [62]. The biologic mechanism for cell necrosis induced by Ti-ions is not yet clear [63].

## 5. Titanium Wear from Instrumentation of Dental Implants for Reparative and Maintenance Procedures

There is no doubt that implant maintenance is required after implant therapy as a standard of care. Many clinicians practice instrumentation of dental implants routinely in order to control and manage peri-implant diseases [64,65,66]. Local antibiotics have also been utilized to treat peri-implantitis [29]. Slow release chemotherapeutic devices with chlorhexidine also seem to have a positive impact on reduction of clinical parameters of peri-implant inflammation [67].

**Figure 14:**
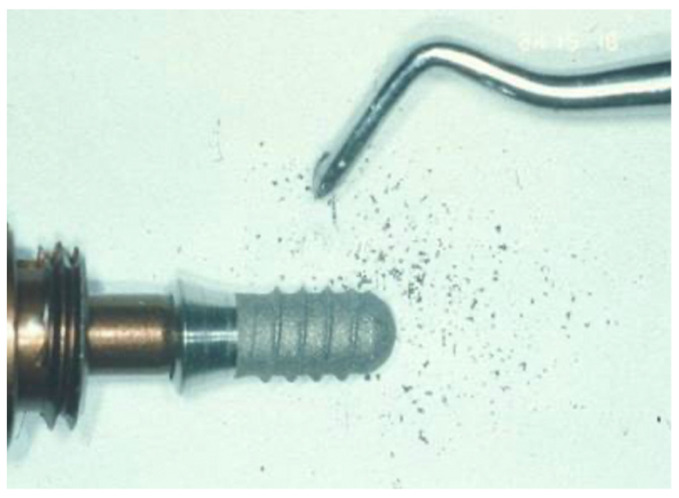
Scaling of the implant surface (Bonefit^®^ implant with TPS-coating) leading to titanium particles removal and damage of the implant surface due to instrumentation. (Courtesy: Prof. Dr. G.-H. Nentwig, Frankfurt, Germany).

Recent studies have shown that mechanical alteration of implant surfaces may lead to negative consequences (Figure 14). The instrumentation of the implants may promote titanium wear, and the resulting debris may lead to local inflammation, increasing the patient’s risk of peri-implant complications, rather than preventing these complications as intended [64].

Peri-implantitis is a feared complication of dental implants as the inflammation and osteolysis surrounding the implant is nearly impossible to reverse [64,68]. Ultrasonic scaling has been used for subgingival debridement to prevent the progression of peri-implantitis. This progression is associated with an increased bioburden leading to an increased host immune response causing destruction of peri-implant tissues [64]. Ultrasonic scaling attempts may lead to further inflammation as opposed to reversing the progression of the condition. Harrel et al. conducted experiments where sandblasted, large grit, acid-etched implants (SLA) were exposed to ultrasonic scaling in vitro. The coolant water from the ultrasonic scaler was analyzed for titanium particles, and the implant surfaces were analyzed for damage to the SLA coating. They concluded that all implants subjected to ultrasonic scaling released titanium particles into the coolant water and all implants showed damage to the SLA surface pattern. The surface damage using ultrasonics has been previously documented in a variety of other studies as well [69,70,71,72,73,74]. These results suggest that the efficacy of ultrasonic scaling of dental implants should be further researched as the resulting titanium wear and implant surface damage may worsen implant health [75].

Pettersson et al. supported the above conclusions with their study [29]. In this study, both mucosa in periodontitis and peri-implantitis was analyzed for the presence of metal particles. Although there was metal debris in periodontitis mucosa, the presence of titanium was far more pronounced in peri-implantitis tissue biopsies. Although these results were not compared to the mucosa surrounding healthy implants, the substantial difference in the presence of titanium in the two inflammatory conditions indicates that titanium wear may worsen peri-implantitis inflammation and lead to a less favorable prognosis for implant survival [29].

Implant manufacturers practice various surface modification strategies in an attempt to increase surface roughness of the implants to increase osseointegration [64,76,77,78,79]. Atomic force microscopy experiments have determined that sandblasted/acid-etched titanium particles have been found to induce a greater inflammatory response and greater differentiation of osteoclasts than lipopolysaccharide from Gram-negative bacteria [64]. In this case, the ability to promote osteoclast differentiation is used to compare inflammatory responses as this is the precursor to bone resorption. Machined discs produce a milder inflammatory response than sandblasted/acid-etched [64].

## 6. Titanium Particles and Immunological Response

Titanium particles shed from the implant surface are immunogenic. Along with dental cement, studies have identified titanium as a predominant foreign body in peri-implantitis biopsies, and these metals are surrounded by inflammatory cells in the tissues [80]. Titanium particles are taken up by macrophages which will release proinflammatory cytokines such as IL-1β, IL-6, and TNF-α [64]. These cytokines are associated with osteoclast activation via the RANK-L/RANK/OPG pathway. This ultimately leads to osteolysis and bone resorption [64]. This process is strongly associated with the development and progression of peri-implantitis. This conclusion is supported by the results of a pilot study conducted by Fretwurst et al. Their group analyzed seven bone samples and five mucosal samples from peri-implantitis sites using synchrotron radiation X-ray fluorescence spectroscopy (SRXRF) and polarized light microscopy (PLM). The biopsies were analyzed for the presence of metals such as iron, titanium, and calcium. Histologic specimens were also analyzed for the presence of macrophages and lymphocytes. The results showed the presence of titanium and iron elements in the biopsies with M1 macrophages and lymphocyte proliferation [81]. These findings, along with a 2018 mini-review from Fretwurst et al. further strengthen the association of metal debris within the peri-implant hard and soft tissues and the progression of peri-implantitis.

Metal debris was first identified as a potential etiologic factor for implant complications in orthopedics and has since then become a growing area of interest in implant dentistry [82]. Specifically, metal debris from implanted devices had been linked to the aseptic loosening and failure of orthopedic implants [83]. Aseptic bone loss is commonly associated with complications in hip replacements as the titanium and zirconium debris, as previously mentioned, is associated with the macrophage inflammatory response [84,85]. This process may lead to undesirable outcomes of osteolysis, and subsequent implant failure [86,87].

Titanium particles shed from implant surfaces range in size from microparticles to NPs. The toxicology of titanium dioxide NPs has been extensively studied. These particles are small enough to be transported systemically throughout the body, and due to their size, they are capable of being internalized by cells, allowing them to interact with organelles and cause intracellular lesions [88,89,90]. Ribeiro et al. conducted a study in 2016 to investigate the interactions between titanium NPs and cells in the biological setting with a goal of better understanding the mechanism of toxicology in bone cells. They found that the NPs could form bio-complexes rich in calcium and phosphate as well as some hydroxyapatite crystalline structures. The consequence of this bio-complex is the internalization of the NPs by osteoblasts. In this context, the bio-complex serves as a mask for the NPs and permits the trafficking of the NPs into and out of cells. Once inside cells, the titanium particles are able to induce DNA damage, possibly via oxidative stress [88,91,92].

Generally, three crystalline phases of titanium dioxide (titania) NPs exist. Anatase (photocatalyst) and rutile are tetragonal, brookite is orthorhombic. The potential toxicity depends on the size and crystalline form. It is important to consider the potential risk from the TiO_2_ NPs in case of “overload” in the body [93].

In 2017, the same research group utilized transmission electron microscopy and graphene liquid cells to further investigate the mechanism by which anatase (TiO_2_) NPs become bio-camouflaged prior to being internalized by cells. It is important to understand the concept of biocompatibility and the issues surrounding the nature of this term. While an implant or medical device may be considered biocompatible as a whole unit, the NPs which are shed from the devices may have different properties which allow them to enter cells in numbers exceeding toxic threshold [94]. The results of this project showed the importance of utilizing graphene liquid cells in imaging as the energy dissipation (conversion of mechanical energy into heat) of this technique preserved the structure of the NPs and proteins. This technique allowed the researchers to confirm the importance of the interaction between titanium NPs and the biological environment when it comes to the internalization of these complexes by bone cells [94].

Hattori et al. further discussed the principles of NP size and toxicology [95]. Their research group analyzed the toxicology of both anatase and rutile TiO_2_ NP, paying careful attention to the crystal form of the particles. Utilizing a lactate dehydrogenase assay and monitoring the uptake of NP by human pleural mesothelial cell cultures, the study found that anatase particles are more readily taken up by cells than rutile particles and are therefore more likely to cause oxidative DNA damage. This indicates that the crystal form of the TiO_2_ NPs plays a key role in toxicology [95]. Several other studies have utilized different assays and cell lines than those used by Hattori et al.; however, they all demonstrate the significance of the crystal form of the TiO_2_ NP and the greater toxicology of the anatase form as opposed to the rutile form [96,97,98,99].

A more critical review of the public health regulations of TiO_2_ was published by Jovanovic, who suggested a reassessment of the safety of TiO_2_ by the relevant governmental agencies as an additive in human food and consider establishing a maximum daily intake as a precaution due to the significant absorption by the gastrointestinal tract and storage in various organs. This might cause damage and modify biochemical metabolic parameters [100].

## 7. Migration of Nanoparticles to Other Tissues after Ingestion following Wear

TiO_2_ NPs are commonly used for medical implant devices due to their high biocompatibility and low toxicity [101,102]. Many studies that support this do not consider the change in properties of these materials when TiO_2_ NPs become detached from the implant surface [103]. NP of titanium can be taken up by an individual in several ways following wear of an implant. The body recognizes these particles as a foreign material and thus an immune response may be initiated [2]. The particles are thus taken up by macrophages in a size-dependent process. The release of inflammatory cytokines as well as macrophage contents further increases local inflammation of the soft and hard tissue surrounding the implant [2,14]. These NPs may also reach other tissues systemically when they are swallowed by the individual in the saliva. Once they are ingested, the particles can be deposited to other tissues and organs, enter the blood and circulatory system, and there is some evidence that these particles are able to cross the blood-brain barrier allowing them to enter the CNS [2,103,104].

The body normally contains 10–20 mg of Ti, and research has shown this level to increase following treatment with medical implant devices [2,105]. Additionally, NP are used in the production of cosmetics and toothpastes, and have been utilized in very low levels in the soil as they have beneficial properties for plant growth and crop production [93,106]. NPs ranging from 10 to 100 nm in size become passive or active targeting systems for drugs in cancer treatment as well as for diagnostic purposes in medicine. The mineral sources of Ti include anatase, rutile, and brookite, each encompassing about 95% TiO_2_ as well as ilmenite (FeOTiO_3_) comprising 40–65% TiO_2_ and leucoxene (Fe_2_O_3_ nTiO_3_) containing more than 65% TiO_2_ [107]. These minerals are generally not soluble. It must be emphasized that in case of high levels of Ti in the plants, Ti may cause phytotoxicity.

Titanium dioxide NP absorption is a complex phenomenon as seen in NPs used in sunscreen for skin protection, which have different scattering and absorption factors within the skin in the different wavelengths of light [108]. However, the heat distribution seems to be the same with the administration of 1% and 5% of titanium NP. When NPs enter the biological environments, they will be in contact with a variety of cells and matrix proteins. This interaction might be critical with respect to their toxicity. Macrophages recognize the NPs as foreign material via specific receptors such as toll-like receptors (TLRs), mannose receptors, Fc receptors, and scavenger receptors. Human macrophages can be stimulated by non-toxic concentrations of titania (TiO_2_), cobalt NPs or zirconia (ZiO_2_) [109]. NPs can interact with mitochondria and cell nuclei, causing NP-related toxicity and oxidative stress. Increased ROS production leads to the activation of mitogen-activated protein kinase (MAPK) and further release of inflammatory cytokines [110,111].

Titanium is transported systemically following uptake by the GI tract as the binding affinity of titanium for transferrin is greater than that of iron [2,112]. Once titanium particles enter the bloodstream, they are concentrated in erythrocytes and transported throughout the body. The primary mechanisms by which metal particles enter cells are diffusion or endocytosis through the plasma membrane. Receptor mediated mechanisms may also be involved with metal particle entry into cells [2]. A study by Schliephake et al. revealed accumulation of titanium particles in the lungs, liver, and kidneys of Göttingen minipigs [113]. This accumulation was far more pronounced in the lungs of the minipigs than the other organs [113]. More recent studies also support this notion of TiO_2_ NPs accumulating in systemic tissues. Weingart et al. found these particles deposited in regional lymph nodes via spectroscopic and energy dispersive x-ray analysis [114]. This means that in addition to ingestion and GI transport, titanium particles may also be systemically transported when they are taken up in the regional lymph nodes via phagocytic immune cells. A relationship has been observed between these particles and systemic diseases such as cardiovascular disease, pulmonary injury, and CNS dysfunction [2,103,115]. However, tissue reactions around metallic implants (different metals were examined) in the rabbit muscle was investigated very early by Laing et al. showing histologically trace elements concentration in the muscle around the metallic implants within a period of 6 months after surgery [116]. The normal concentrations were reported previously by Ferguson et al. and these and were not related to wear [117,118].

Corrosion of varying degree was identified close to the metal surface forming a pseudo-membrane with varying thicknesses. The thickness was the lowest around titanium, tungsten and zirconium compared to the increased thickness around iron, manganese, cobalt, molybdenum, chromium, and vanadium. Interestingly, the pseudo membrane around titanium or titanium alloys was consistently thin and around zirconia implants the results were not satisfactory since the thickness was related to the consistency of the zirconia alloys.

Additional studies in baboons using titanium-based prosthetic segmental replacements in long bones showed a six-fold increase of titanium in the implanted group, but no difference in the serum of the animals. The authors also found an increase in the titanium concentration in the lungs, spleen, and regional lymph nodes up to 12 months after implantation. Serum chemistry and hematological analysis did not show any statistical differences in any of the tested electrolytes or complete blood counts and the data were similar to the normal baboons without surgery [119].

Palazzo et al. utilized histological analysis to show the release of metals from osteosynthesis implants even in bone after the removal of the plates where radiological findings were no longer found [17]. The authors concluded that metal wear could also take place during the actual fixation of the implant into the bone, with the highest concentration of metal found close to the osteosynthesis or implant fixation. However, migration of the metal particles was found as far as 2.5 cm away from the implant in the study. More recent studies showed migration of titanium ions to oral mucosa close to metal restorations as accumulated trace metal elements in biopsy specimens from the oral mucosa and around dental implants [120,121].

There is a growing concern regarding the potential of titanium NP to enter the central nervous system (CNS). Their small size and biocompatibility enable them to cross the blood-brain barrier [103]. These particles may also alter the permeability of the barrier to allow more particles to diffuse across [103,104]. Once the nanoparticles reach the CNS, they have the potential to induce inflammation of neurons and glial cells [103,122,123]. Inductively coupled plasma mass spectrometry has been used in various experiments to determine the presence of Ti experimentally in the brain. A relationship has been observed between titanium nanoparticles in the CNS and neurodegenerative disorders such as Alzheimer’s disease [2,103]. Aside from the blood-brain barrier pathway to the CNS, there is also concern that titanium nanoparticles may enter the CNS via the placental barrier [103,124]. This implies that there is a possibility that titanium NPs may be present in the CNS of the fetus of a mother with titanium implant devices [103].

TiO_2_ NPs have previously been thought to be poorly soluble and possess low toxicity, but recent studies have explored other properties of these NP, which play a role in their toxicity to various systems. The key structural features of TiO_2_ NP in this regard are size, shape, coating, and crystal phase [115]. It has been found that these properties as they pertain to TiO_2_ NP facilitate the accumulation of the particles in the interstitium of lung tissue if they are inhaled [115]. Additionally, thin sections from the liver of rats showed a distribution of titanium in after either repeated oral or single intravenous doses of titanium citrate. Therefore, soluble titanium is retained in the organs after i.v. exposure and the distribution seem to be different than those of TiO_2_ NP. Specifically, the titanium citrate soluble metal can be quickly eliminated by the kidneys, but titanium metal can gradually accumulate in the liver [125].

The current crystalline phases of titanium on the implant surface play a significant role in the biomaterial sciences for improvement of the osseointegration and clinical applications. The modern nanotechnology plays a key role in the identification and elimination of side effects from the scientific metallurgic reactions of the titanium in order to control hypersensitivity of patients to the titanium as material. Plaque accumulation in the oral cavity reduces the corrosion resistance of the titanium surface [126]. Growth of the oral bacterium *Actinomyces naeslundii* with pure titanium within seven days has been shown. This bacterial growth depends on the surface energy and TiO_2_ crystalline phase, which influence the release of bone morphogenetic proteins-2 (BMP-2) when nanotubes have a diameter up to 120 nm or when the diameters are smaller (80 nm) control the expression of pre-inflammatory mediators, such as cytokines and chemokines [125,126,127,128,129]. In addition, the titanium surface area and chemical composition significantly influence the cell response especially when surfaces are in the nanoscale level. Material sciences create today and continue to focus on the future development of TiO_2_ nanocrystals with high stability, especially since TiO_2_ NPs release from the implant surfaces induce pro-inflammatory responses, cell surface damage and cytotoxicity.

## 8. Conclusions

The present literature review highlights the significance of titanium wear from implant surfaces. It is concluded that titanium wear likely begins at the time of implant placement and continues under forces of mastication. Maintenance and reparative procedures aimed towards preventing the progression of peri-implant mucositis to peri-implantitis may also increase the amount of titanium particles released from the implant surfaces. The size of the titanium particles is critical to their ability to be immunogenic, and there is strong evidence that anatase (TiO_2_ nanoparticles) can cause local inflammation. Additionally, these particles may be ingested from the oral cavity and eventually cause systemic inflammatory reactions since animal studies show translocation of NPs in different organs. Future research in this field is critical to better understand the full extent of the toxicity of titanium dioxide NP, as well as how great of a role these particles play in inducing inflammation associated with peri-implantitis and implant failure.

## Abbreviations

NP	Nanoparticles
CP	Commercially Pure
SRXRF	Synchrotron radiation X-ray fluorescence spectroscopy
PLM	Polarized light microscopy
ROS	Reactive oxygen species
SLA	Sandblasted, large grit, acid-etched
TLR	Toll-like receptors
MAPK	Mitogen-activated protein kinase
CNS	Central nervous system
BMP-2	Bone morphogenetic proteins-2
